# The discriminating ability of multiple body composition measures in assessing cardiorespiratory fitness in Chinese adolescents aged 12–18

**DOI:** 10.3389/fnut.2026.1842738

**Published:** 2026-05-26

**Authors:** Bo Huang, Jianping Xiong, Guangxin Chai, Li Xiong

**Affiliations:** 1 School of Physical Education, Shangrao Normal University, Shangrao, Jiangxi, China; 2 School of Physical Education, Jiangxi University of Finance and Economics, Nanchang, Jiangxi, China; 3 School of Physical Education & Health, Jiangxi Science and Technology Normal University, Nanchang, Jiangxi, China; 4 School of Physical Education & Health, Nanchang Institute of Science & Technology, Nanchang, Jiangxi, China

**Keywords:** adolescents, associations, body composition, cardiorespiratory fitness, identification ability

## Abstract

**Background:**

Body mass index (BMI) and waist circumference (WC) are widely used as traditional indicators of body composition. However, the relationship between the weight-adjusted waist index (WWI), the a body shape index (ABSI), and the body roundness index (BRI)—as newer derived indices—and cardiopulmonary fitness (CRF) in adolescents remains unclear. The purpose of this study is to discriminating ability the effectiveness of BMI, WC, WWI, ABSI, and BRI in identifying cardiorespiratory fitness among Chinese adolescents. The aim is to provide a reference and basis for improving their cardiorespiratory fitness levels.

**Methods:**

This study conducted a cross-sectional assessment of 45,917 adolescents aged 12–18 years in mainland China, evaluating their height, weight, WC, 20-meter shuttle run test (20 m-SRT), as well as family background and lifestyle factors. Using univariate analysis of variance, Spearman’s rank correlation, multivariate logistic regression, and ROC analysis, we compared the associations between BMI, WC, WWI, ABSI, and BRI and cardiorespiratory fitness, as well as the effectiveness of these indices in identifying adolescents with insufficient cardiorespiratory fitness.

**Results:**

The mean values for BMI, WC, WWI, ABSI, and BRI among Chinese adolescents were (20.19 ± 3.43) kg/m^2^, (69.7 ± 10.05) cm, (9.41 ± 1.03), (0.06 ± 0.01), and (2.06 ± 0.92), respectively. The average 20-meter SRT score for adolescents was (40.63 ± 18.35) laps. ROC analysis showed that WWI had the highest effectiveness in identifying insufficient cardiorespiratory fitness among girls, with an AUC of 0.73 (0.72–0.73), followed by BRI (0.72). For boys, BRI (0.66) and WWI (0.65) performed best in identifying insufficient cardiorespiratory fitness. Overall, WWI and BRI demonstrated the best identification performance (both with an AUC of 0.69), followed by WC (0.65), while ABSI (0.60) and BMI (0.56) were relatively weaker.

**Conclusion:**

Various body composition measures in Chinese adolescents demonstrate a certain ability to identify insufficient cardiorespiratory fitness; however, there are significant differences in predictive performance based on sex and between different measures. Future interventions targeting cardiorespiratory fitness in adolescents should take into account sex differences in the influence of various body composition measures.

## 1 Introduction

As modern lifestyles continue to evolve, the cardiopulmonary fitness of adolescents has been declining year by year, posing serious negative impacts on their physical and mental health and emerging as a public health concern shared by countries around the world (1). Studies show that the cardiorespiratory fitness of adolescents in European countries has been on a steady decline over the past few decades, a trend linked to rising obesity rates (2). Studies also show that cardiovascular fitness among Swedish adolescents has been on a downward trend over the past few decades, posing a serious threat to their health (3). Similarly, China, as a developing country, is no exception. Studies show that over the past few decades, the cardiorespiratory fitness of Chinese adolescents has declined significantly; the current average maximum oxygen uptake is approximately 46.47 mL/kg/min, which has serious negative implications for academic performance and mental health (4). Cardiovascular fitness, as a core component of adolescents’ physical health, has a significant impact on their academic performance, mental health, physical health, and future health in adulthood (5). Research shows that high levels of cardiorespiratory fitness during adolescence are closely associated with body composition, blood pressure, metabolic health, and cognitive function, and have far-reaching implications for work capacity, chronic diseases, and quality of life in adulthood (6, 7). A meta-analysis showed that, compared with the lowest third of participants in terms of cardiorespiratory fitness, those in the highest third had a 45% lower risk of all-cause mortality (8). Other studies have shown that when CRF is analyzed continuously using metabolic equivalents (METs) as a unit of measurement, a 1-MET increase is associated with an average 11–14% reduction in the risk of all-cause mortality, and this association remains consistent across sex (9). The study also found that poor cardiorespiratory fitness during adolescence leads to a sustained increase in the risk of developing various chronic diseases in adulthood, posing a serious threat to future health (10). Research also shows that there is a significant positive correlation between adolescents’ cardiorespiratory fitness and academic performance; those with higher cardiorespiratory fitness tend to achieve better grades in mathematics and English, which may be related to the significant link between cardiorespiratory fitness and executive function in the brain (11, 12).

However, there are many factors that influence cardiovascular and respiratory fitness levels, such as body composition, dietary habits, and physical activity patterns (13, 14). First, physical activity levels among adolescents are steadily declining, with a particularly marked decrease in moderate-to-vigorous physical activity, which has a detrimental effect on their cardiopulmonary fitness (15). A study of Japanese adolescents found that for every 5-min decrease in physical activity, their 20-meter shuttle run test (20-m SRT) scores—a measure of cardiorespiratory fitness—decreased significantly: by 1.2 laps for boys and 2.3 laps for girls (16). Second, the increasing duration of sedentary and screen-based activities among adolescents has led to reduced muscle strength and obesity, and has also had a negative impact on their cardiorespiratory fitness (17). Research has found a significant negative correlation between increased screen time among adolescents and decreased cardiorespiratory fitness (18). At the same time, changes in dietary habits and the consumption of foods high in sugar, salt, and fat can also lead to obesity, which has a negative impact on adolescents’ cardiorespiratory fitness (19). Given the critical role and significance of cardiopulmonary fitness in adolescents’ mental and physical health, as well as its association with body composition, it is particularly important and practical to use simpler and more effective body composition indicators to identify deficiencies in cardiopulmonary fitness.

In recent years, the global prevalence of obesity among adolescents has been on the rise, while their cardiorespiratory fitness has been declining, giving rise to widespread public health concerns. Numerous studies have shown that body composition is a key factor influencing cardiorespiratory fitness in adolescents and warrants close attention and priority (20). Studies have shown that BMI and waist circumference (WC)—common indicators of body composition—are significantly associated with cardiorespiratory fitness. Increases in both BMI and WC are associated with a decline in cardiorespiratory fitness, which can have serious negative consequences for physical health (21). In addition, studies have shown that higher body fat levels and central obesity—as measured by WC and waist-to-height ratio (WHtR)—are significantly associated with lower cardiorespiratory fitness (22). Furthermore, this negative correlation has the same effect on both boys and girls, though the specific patterns of influence may differ. For example, one study showed that, after adjusting for the effect of height, body fat percentage had a greater impact on girls than on boys (23). Other studies have shown that there is an inverted U-shaped relationship between WWI and cardiorespiratory fitness, indicating a significant association between the two (24). Other studies have shown that the ABSI index, which reflects body composition, is significantly associated with cardiorespiratory fitness. This may be because the ABSI provides a more comprehensive reflection of visceral fat in the body, thereby explaining the significant association (25). This indicates that there has been a growing body of research examining the relationship between body composition indices and cardiorespiratory fitness in adolescents. However, as attention to body composition in adolescents has increased, several new indices reflecting body composition—such as WWI, ABSI, and BRI—have emerged in recent years. It remains unclear how these indices compare to traditional measures like BMI and WC in terms of their ability to predict cardiorespiratory fitness. Furthermore, while past studies have analyzed the association between various body composition indices and cardiorespiratory fitness, the indices used in these analyses have limitations, and the results have been inconsistent. For example, a study on adolescents showed that BMI and WWI are significantly associated with cardiorespiratory fitness (26, 27). However, another study of a different subpopulation found that the classic association between body composition and CRF may not hold; for example, no significant association was found between body composition and CRF among adolescents who regularly participated in organized physical training (28). Previous studies on the relationship between indicators of body composition and cardiorespiratory fitness have yielded inconsistent findings, warranting further in-depth research and analysis. At the same time, due to their limited sample sizes, these studies lack representativeness. As research progresses, ABSI—a novel body composition indicator proposed by Krakauer—is associated with multiple indicators of physical health, and appears to be more closely correlated with these indicators than BMI or waist circumference (29, 30). However, the results were not entirely consistent. Studies have shown that BMI is a relatively stronger predictor of cardiovascular disease than the ABSI, meaning that the ABSI does not perform better in identifying the onset of cardiovascular disease (31).

It is worth noting that there is currently limited research on the relationship between new body composition indices such as ABSI and BRI and cardiorespiratory fitness; instead, most studies have focused on physiological indicators such as cardiovascular disease (32, 33). Currently, it remains unclear whether there are differences in the ability of traditional BMI and WC measures versus their derived indices—WWI, ABSI, and BRI—to assess cardiorespiratory fitness in Chinese adolescents. Given the limitations of previous studies, this study employs a large national sample to analyze the associations between the body composition indices BMI, WC, WWI, ABSI, and BRI and cardiorespiratory fitness in Chinese adolescents. This study aims to better identify the body composition indicators that influence adolescents’ cardiorespiratory fitness, thereby providing a reference and guidance for improving and intervening in the cardiorespiratory fitness levels of Chinese adolescents.

## 2 Methods

### 2.1 Participants

**Figure 1:**
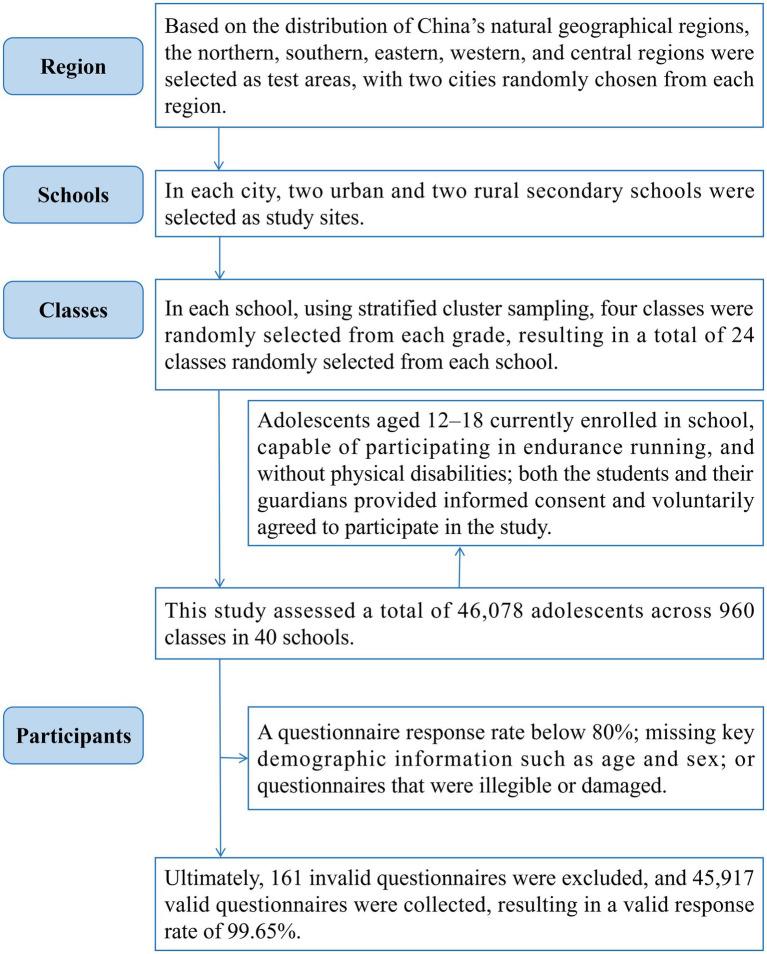
Sampling process for Chinese adolescents aged 12–18.

This study employed a stratified cluster sampling method to conduct a cross-sectional assessment of height, weight, WC, 20-meter SRT, family background, and lifestyle among 45,917 adolescents aged 12–18 in mainland China. The participant selection process was conducted in three stages. First, based on the distribution of China’s natural geographical regions, the northern, southern, eastern, western, and central regions were selected as test areas, with two cities randomly chosen from each region. Second, in each city, two urban and two rural secondary schools were selected as study sites. Third, in each school, using stratified cluster sampling, four classes were randomly selected from each grade, resulting in a total of 24 classes randomly selected from each school. This study assessed a total of 46,078 adolescents across 960 classes in 40 schools. The inclusion criteria were: adolescents aged 12–18 currently enrolled in school, capable of participating in endurance running, and without physical disabilities; both the students and their guardians provided informed consent and voluntarily agreed to participate in the study. The exclusion criteria for this study were: a questionnaire response rate below 80%; missing key demographic information, such as age and sex; or questionnaires that were illegible or damaged. Ultimately, 161 invalid questionnaires were excluded, and 45,917 valid questionnaires were collected, resulting in a valid response rate of 99.65%. The specific sampling process for the study participants is shown in Figure 1.

This study was conducted in accordance with the principles of the Declaration of Helsinki. Written assent was obtained from parents/guardians. The participants also signed up for the study on their own. Written informed consent for participation in this study was provided by the participants or the participants’ legal guardians/next of kin’. This study was approved by the Ethics Committee of Jiangxi Science and Technology Normal University (IRB-JXSTNU-2022003).

### 2.2 Body composition assessment

#### 2.2.1 Body mass index (BMI)

BMI is calculated indirectly based on a participant’s height and weight. BMI = weight (kg)/height (m)^2^. In this study, height and weight were measured in accordance with the guidelines of the China National Survey on Students’ Constitution and Health (CNSSCH) (34). Height measurements are accurate to 0.1 centimeters. Weight measurements are accurate to 0.1 kilograms. The height and weight measurement devices are calibrated daily to ensure accuracy before measurements are taken. Measurements are taken using the Wanqing RGZ-160 height and weight scale.

#### 2.2.2 Waist circumference (WC)

The evaluation method for WC is based on the methodology specified in the China National Survey on Students’ Constitution and Health (CNSSCH) (34). Participants are asked to wear as light and loose-fitting clothing as possible before the assessment. Depending on the participant’s sex, the assessment will be conducted by a staff member of the same sex. Measurements are recorded to the nearest 0.1 centimeter. A nylon tape measure is used for the assessment, which is conducted with the participant standing. The tape measure is wrapped parallel to the abdomen, 1 cm above the navel, and the WC measurement is recorded.

#### 2.2.3 Weight-adjusted waist index (WWI)

The WWI is calculated using a fixed formula based on the participant’s weight and waist circumference. WWI=WC(cm)/ $\sqrt{\text{Weight}\;(\mathrm{kg})}$ .

#### 2.2.4 A body shape index (ABSI)

The ABSI is calculated using the participant’s BMI and WC. The formula for the ABSI is WC/(BMI^2^/3 × height^1^/^2^) (29).

#### 2.2.5 Body roundness index (BRI)

The calculation for BRI is 364.2–365.5*(1-[WC(m)/2π]^2^/[0.5*height(m)]^2^)½ (35).

### 2.3 Assessment of cardiopulmonary fitness (CRF)

In this study, cardiopulmonary fitness was assessed using the 20-meter SRT, a test commonly used internationally (36). The 20-m SRT assessment uses an audio signal to control the participant’s running pace. Participants run back and forth over a 20-meter distance, starting at an initial speed of 8.0 km/h, which increases by 0.5 km/h per minute across 21 levels, for a maximum of 247 laps. Participants must reach the end line and touch the line before or at the same time as the audio “beep” sounds. The test is terminated if a participant fails to keep pace twice in a row. Each 20-meter run is recorded as one lap. In this study, the number of laps completed by each participant after the test is recorded as their individual cardiorespiratory fitness score. After stratifying participants by age and sex, those with an average 20-m SRT score below one standard deviation were defined as having inadequate or substandard cardiorespiratory fitness.

### 2.4 Covariates

The covariates included in this study were place of residence, father’s education, mother’s education, monthly household income, and SSB consumption (37, 38). Place of residence is categorized into Large and medium-sized cities, Small cities, and Rural areas. Father’s education and Mother’s education are categorized into primary school, middle school, high school, and college or above. Monthly household income is categorized into <2,000 yuan/month, 2,001–5,000 yuan/month, 5,001–8,000 yuan/month, and >8,000 yuan/month. SSB consumption was calculated based on the frequency of SSB consumption by participants over the past 7 days, with each consumption serving as a reference standard of one 330-milliliter can. In this study, consumption was categorized as ≤2 times/week, 3–4 times/week, and ≥5 times/week.

### 2.5 Quality control

When participants undergo assessments of height, weight, and WC, they are asked to wear as little and thin clothing as possible and to remove their shoes before the assessment. Participants are required to empty their bowels and bladder before the assessment. Assessors calibrate the measurement instruments daily to ensure the accuracy of the assessments. Assessments of covariates are conducted using paper-based questionnaires. Staff explain the purpose and requirements of the assessment to participants, distribute the questionnaires on-site, and collect them immediately afterward. Participants complete the questionnaires independently based on their actual circumstances, free from the influence or restrictions of others. If participants have any questions during the assessment process, they may ask the staff for clarification.

### 2.6 Statistical analysis

The results for the participants in this study are presented as either continuous or categorical variables. Continuous variables, such as height, weight, BMI, WC, and 20-m SRT, are presented as mean and standard deviation. Categorical variables, including place of residence, father’s education, mother’s education, monthly household income, and SSBs, are presented as percentages (%). Prior to analysis, the data were tested for normality, and all data points were found to follow a normal distribution. Pearson correlation analysis was used to examine the relationships between 20-m SRT and BMI, WC, WWI, ABSI, and BRI, with r values reported for each. After stratifying by age and sex, participants with 20-m SRT scores below one standard deviation were classified as having insufficient or suboptimal cardiorespiratory fitness (39). After stratification by age and sex, BMI, WC, WWI, ABSI, and BRI were divided into quartiles, resulting in four groups: Q1, Q2, Q3, and Q4. The chi-square test was used to compare the percentages of participants with insufficient cardiopulmonary fitness across the quartile groups. The association between the 20-m SRT and BMI, WC, WWI, ABSI, and BRI in adolescents was analyzed using binary logistic regression. The presence or absence of suboptimal cardiorespiratory fitness was used as the dependent variable, and the quartiles of BMI, WC, WWI, ABSI, and BRI were used as independent variables in the logistic regression analysis. Model 1 did not adjust for covariates, while Model 2 adjusted for age, place of residence, father’s education, mother’s education, monthly household income, and SSB in addition to the variables in Model 1. The analysis results reported OR values and 95% CIs. We also performed multicollinearity diagnostics on multiple covariates; both the tolerance and VIF values met the requirements, and there were no issues of multicollinearity among the variables. To further analyze the validity of body composition indicators (BMI, WC, WWI, ABSI, and BRI) in identifying cardiopulmonary fitness. This study employed ROC analysis. With the presence or absence of cardiopulmonary fitness deficits (i.e., meeting or failing to meet standards) as the status variable, and BMI, WC, WWI, ABSI, and BRI as predictor variables, ROC analysis was conducted. The effectiveness of body composition indices in identifying cardiopulmonary fitness was evaluated based on the area under the curve (AUC) and 95% confidence interval (CI). Statistical processing and analysis of the data were performed using SPSS 25.0 software. A *p*-value of <0.05 was considered statistically significant.

## 3 Results

This study assessed body composition and 20-m SRT in 45,917 Chinese adolescents aged 12–18 years. The mean age of the participants was (15.12 ± 1.88) years. The mean values for BMI, waist circumference, WWI, ABSI, and BRI among the 12–18-year-old adolescents in this study were (20.19 ± 3.43) kg/m^2^, (69.7 ± 10.05) cm, (9.41 ± 1.03), (0.06 ± 0.01), and (2.06 ± 0.92), respectively. The mean 20-m SRT for participants in this study was (40.63 ± 18.35) laps. A comparison of the various indicators between participants who met and those who did not meet the 20-meter SRT standard is shown in Table 1.

**Table 1:** A comparison of characteristics associated with meeting the 20-m SRT standard among Chinese adolescents aged 12–18.

Categorization	Inadequate or substandard 20-m SRT		Total	Chi-square/*t*-value	*p*-value
	Yes	No			
*N*	21,365	24,552	45,917		
Age	15.14 ± 1.87	15.11 ± 1.89	15.12 ± 1.88	1.322	0.186
Height	166.03 ± 8.8	165.06 ± 9.00	165.51 ± 8.92	11.610	<0.001
Weight	54.82 ± 10.93	56.35 ± 12.98	55.64 ± 12.09	13.552	<0.001
BMI	19.79 ± 3.04	20.55 ± 3.71	20.19 ± 3.43	23.881	<0.001
Waist circumference	66.86 ± 8.61	72.16 ± 10.54	69.7 ± 10.05	58.464	<0.001
WWI	9.09 ± 0.91	9.69 ± 1.04	9.41 ± 1.03	65.340	<0.001
ABSI	0.06 ± 0.01	0.06 ± 0.01	0.06 ± 0.01	35.952	<0.001
BRI	1.75 ± 0.72	2.32 ± 0.99	2.06 ± 0.92	69.014	<0.001
20 m SRT	54.89 ± 15.23	28.21 ± 9.84	40.63 ± 18.35	225.615	<0.001
Sex				0.587	0.444
Boys	10,612(49.7)	12,107(49.3)	22,719(49.5)		
Girls	10,753(50.3)	12,445(50.7)	23,198(50.5)		
Place of residence				120.585	<0.001
Large and medium-sized cities	8,573(40.1)	9,591(39.1)	18,164(39.6)		
Small city	7,144(33.4)	9,306(37.9)	16,450(35.8)		
Countryside	5,648(26.4)	5,655(23.0)	11,303(24.6)		
Father’s education				30.190	<0.001
Primary School	2,455(11.5)	2,834(11.5)	5,289(11.5)		
Middle School	7,365(34.5)	8,953(36.5)	16,318(35.5)		
High School	7,337(34.3)	8,339(34.0)	15,676(34.1)		
College or above	4,208(19.7)	4,426(18.0)	8,634(18.8)		
Mother’s education				37.281	<0.001
Primary School	3,915(18.3)	4,203(17.1)	8,118(17.7)		
Middle School	7,037(32.9)	8,718(35.5)	15,755(34.3)		
High School	6,974(32.6)	7,878(32.1)	14,852(32.3)		
College or above	3,439(16.1)	3,753(15.3)	7,192(15.7)		
Monthly household income				120.350	<0.001
<2000	2,636(12.3)	2,893(11.8)	5,529(12.0)		
2001–5,000	7,117(33.3)	9,060(36.9)	16,177(35.2)		
5,001–8,000	6,403(30.0)	7,544(30.7)	13,947(30.4)		
>8,000	5,209(24.4)	5,055(20.6)	10,264(22.4)		
SSB				5.395	0.067
≤2 times/week	6,956(32.6)	8,242(33.6)	15,198(33.1)		
3–4 times/week	11,193(52.4)	12,696(51.7)	23,889(52.0)		
≥5 times/week	3,216(15.1)	3,614(14.7)	6,830(14.9)		

SSB, sugar-sweetened beverage.

The results in Table 2 show that BMI, WC, WWI, ABSI, and BRI all exhibited a significant negative correlation with 20-m SRT scores (*p* < 0.001), indicating that the greater the accumulation of body fat in adolescents, the poorer their cardiorespiratory fitness performance. Regarding sex differences, among girls, WC showed the strongest negative correlation with 20-m SRT (*r* = −0.408), followed by BRI (*r* = −0.426); for boys, BRI showed the strongest correlation (*r* = −0.372), followed by WWI (*r* = −0.339). Overall, BRI and WWI demonstrated strong negative associations with the 20-m SRT (*r* = −0.334, −0.321), further illustrating that abdominal fat distribution has a particularly pronounced negative impact on adolescents’ cardiorespiratory fitness.

**Table 2:** Correlation analysis of BMI, WC, WWI, ABSI, and BRI with the 20-m SRT in Chinese adolescents aged 12–18.

Sex	BMI	WC	WWI	ABSI	BRI
Boys (*n* = 22,719)					
*r*-value	−0.155	−0.279	−0.339	−0.175	−0.372
*P*-value	<0.001	<0.001	<0.001	<0.001	<0.001
Girls (*n* = 23,198)					
*r*-value	−0.134	−0.408	−0.388	−0.223	−0.426
*P*-value	<0.001	<0.001	<0.001	<0.001	<0.001
Total (*n* = 45,917)					
*r*-value	−0.105	−0.206	−0.321	−0.188	−0.334
*P*-value	<0.001	<0.001	<0.001	<0.001	<0.001

Table 3 presents a univariate analysis of the relationship between various body composition indices and the prevalence of failure to meet the 20-m SRT standard among Chinese adolescents aged 12–18 years. Overall, it can be observed that as the quartiles of BMI, WC, WWI, ABSI, and BRI increase, the prevalence of failure to meet the 20-m SRT standard among adolescents also shows an upward trend (*p* < 0.001). The same trend is observed across different sex groups.

**Table 3:** Univariate comparison of different body composition measures and the prevalence of 20-meter SRT inadequacy among Chinese adolescents aged 12–18.

Quartile	BMI	WC	WWI	ABSI	BRI
Boys					
Q1[*n*, (%)]	2,708(50.8)	1751(42.4)	2059(37.4)	2,806(46.8)	1815(36.0)
Q2[*n*, (%)]	2,341(46.0)	1792(42.7)	2,424(45.4)	2,977(49.8)	2,503(46.8)
Q3[*n*, (%)]	2,702(48.8)	3,382(49.7)	3,142(56.7)	2,958(56.8)	2,908(51.2)
Q4[*n*, (%)]	4,356(64.4)	5,182(68.2)	4,482(70.8)	3,366(60.8)	4,881(73.5)
*t*-value	504.208	1092.854	1496.795	283.281	1795.816
*P*-value	<0.001	<0.001	<0.001	<0.001	<0.001
Girls					
Q1[*n*, (%)]	3,092(51.4)	2,767(34.8)	1,634(27.0)	1890(36.2)	1810(29.8)
Q2[*n*, (%)]	3,193(49.8)	3,228(50.0)	2,966(47.9)	2,888(45.9)	2,901(46.3)
Q3[*n*, (%)]	3,054(51.2)	3,931(68.3)	3,878(66.8)	3,888(63.6)	3,719(62.8)
Q4[*n*, (%)]	3,106(64.7)	2,519(83.1)	3,967(77.0)	3,779(67.8)	4,015(81.3)
*t*-value	300.284	2729.517	3339.135	1479.773	3240.414
*P*-value	<0.001	<0.001	<0.001	<0.001	<0.001
Total					
Q1[*n*, (%)]	5,800(51.1)	4,518(37.4)	3,693(32.0)	4,696(41.9)	3,625(32.6)
Q2[*n*, (%)]	5,534(48.1)	5,020(47.1)	5,390(46.8)	5,865(47.8)	5,404(46.5)
Q3[*n*, (%)]	5,756(50.0)	7,313(58.3)	7,020(61.8)	6,846(60.5)	6,627(57.1)
Q4[*n*, (%)]	7,462(64.5)	7,701(72.4)	8,449(73.6)	7,145(64.3)	8,896(76.8)
*t*-value	781.831	3078.21	4543.439	1512.897	4766.744
*P*-value	<0.001	<0.001	<0.001	<0.001	<0.001

Q, Quartile.

Using whether a person meets the standard for the 20-meter SRT as the dependent variable and the quartiles of BMI, WC, WWI, ABSI, and BRI as independent variables. Model 1 was unadjusted for covariates, while Model 2 adjusted for age, place of residence, father’s education, mother’s education, monthly household income, and SSB consumption in addition to the variables in Model 1. Overall, using the Q1 quartile of BMI, WC, WWI, ABSI, and BRI as the reference group, the Q4 quartile had the highest of failing the 20-meter SRT, with OR values of 1.75, 4.48, 5.95, 2.51, and 6.88, respectively. These differences were statistically significant (*p* < 0.001). Analysis results by sex are shown in Table 4.

**Table 4:** Binary logistic regression analysis of the relationship between various body composition indices and the prevalence of substandard 20-m SRT results among Chinese adolescents aged 12–18.

Quartile	BMI		WC		WWI		ABSI		BRI	
	Model 1	Model 2	Model 1	Model 2	Model 1	Model 2	Model 1	Model 2	Model 1	Model 2
Boys										
1(Reference)	1.00	1.00	1.00	1.00	1.00	1.00	1.00	1.00	1.00	1.00
2[OR(95%CI)]	0.83(0.77 ~ 0.89) ^c^	0.83(0.77 ~ 0.89) ^c^	1.01(0.93 ~ 1.10)	1.02(0.94 ~ 1.11)	1.39(1.29 ~ 1.50) ^c^	1.41(1.30 ~ 1.52) ^c^	1.13(1.05 ~ 1.21) ^b^	1.14(1.06 ~ 1.22) ^c^	1.57(1.45 ~ 1.70) ^c^	1.57(1.45 ~ 1.70) ^c^
3[OR(95%^c^I)]	0.92(0.86 ~ 1.00) ^a^	0.93(0.86 ~ 1.00)	1.34(1.24 ~ 1.45) ^c^	1.36(1.25 ~ 1.47) ^c^	2.19(2.03 ~ 2.36) ^c^	2.21(2.04 ~ 2.38) ^c^	1.49(1.39 ~ 1.61) ^c^	1.51(1.40 ~ 1.63) ^c^	1.87(1.73 ~ 2.02) ^c^	1.88(1.74 ~ 2.03) ^c^
4[OR(95%^c^I)]	1.76(1.63 ~ 1.89) ^c^	1.76(1.64 ~ 1.9) ^c^	2.90(2.69 ~ 3.14) ^c^	2.95(2.73 ~ 3.19) ^c^	4.06(3.76 ~ 4.38) ^c^	4.08(3.78 ~ 4.41) ^c^	1.77(1.64 ~ 1.90) ^c^	1.78(1.65 ~ 1.91) ^c^	4.93(4.56 ~ 5.34) ^c^	4.97(4.59 ~ 5.38) ^c^
Girls										
1(Referen^c^e)	1.00	1.00	1.00	1.00	1.00	1.00	1.00	1.00	1.00	1.00
2[OR(95%^c^I)]	0.94(0.87 ~ 1.01)	0.94(0.87 ~ 1.00)	1.87(1.75 ~ 2.00) ^c^	1.89(1.76 ~ 2.02) ^c^	2.49(2.30 ~ 2.68) ^c^	2.49(2.31 ~ 2.68) ^c^	1.49(1.38 ~ 1.61) ^c^	1.50(1.39 ~ 1.62) ^c^	2.03(1.89 ~ 2.19) ^c^	2.04(1.89 ~ 2.20) ^c^
3[OR(95%^c^I)]	0.99(0.92 ~ 1.07)	0.99(0.92 ~ 1.07)	4.04(3.76 ~ 4.34) ^c^	4.07(3.78 ~ 4.37) ^c^	5.43(5.01 ~ 5.87) ^c^	5.46(5.04 ~ 5.91) ^c^	3.07(2.85 ~ 3.32) ^c^	3.08(2.86 ~ 3.33) ^c^	3.98(3.69 ~ 4.29) ^c^	3.99(3.70 ~ 4.31) ^c^
4[OR(95%^c^I)]	1.73(1.60 ~ 1.87) ^c^	1.73(1.60 ~ 1.87) ^c^	9.24(8.31 ~ 10.27) ^c^	9.31(8.37 ~ 10.35) ^c^	9.05(8.30 ~ 9.87) ^c^	9.05(8.30 ~ 9.87) ^c^	3.70(3.42 ~ 4.01) ^c^	3.71(3.43 ~ 4.02) ^c^	10.22(9.34 ~ 11.19) ^c^	10.22(9.34 ~ 11.19) ^c^
Total										
1(Referen^c^e)	1.00	1.00	1.00	1.00	1.00	1.00	1.00	1.00	1.00	1.00
2[OR(95%^c^I)]	0.89(0.84 ~ 0.94) ^c^	0.89(0.84 ~ 0.93) ^c^	1.49(1.42 ~ 1.57) ^c^	1.50(1.42 ~ 1.58) ^c^	1.87(1.77 ~ 1.97) ^c^	1.88(1.78 ~ 1.98) ^c^	1.27(1.21 ~ 1.34) ^c^	1.28(1.21 ~ 1.35) ^c^	1.80(1.71 ~ 1.90)	1.81(1.71 ~ 1.91) ^c^
3[OR(95%^c^I)]	0.96(0.91 ~ 1.01)	0.96(0.91 ~ 1.01)	2.34(2.22 ~ 2.46) ^c^	2.36(2.24 ~ 2.48) ^c^	3.45(3.27 ~ 3.64) ^c^	3.47(3.29 ~ 3.66) ^c^	2.12(2.01 ~ 2.24) ^c^	2.13(2.02 ~ 2.25) ^c^	2.76(2.61 ~ 2.91)	2.77(2.62 ~ 2.92) ^c^
4[OR(95%^c^I)]	1.74(1.65 ~ 1.84) ^c^	1.75(1.66 ~ 1.84) ^c^	4.40(4.16 ~ 4.65) ^c^	4.48(4.24 ~ 4.74) ^c^	5.93(5.60 ~ 6.28) ^c^	5.95(5.62 ~ 6.30) ^c^	2.50(2.37 ~ 2.64) ^c^	2.51(2.38 ~ 2.65) ^c^	6.84(6.45 ~ 7.26)	6.88(6.48 ~ 7.29) ^c^

^a^*P* < 0.05, ^b^*P* < 0.01, ^c^*P* < 0.001.

The ROC analysis results show that the AUC values for all body composition indicators were above 0.50, indicating that they all possess a certain ability to identify insufficient cardiorespiratory fitness; however, there were significant differences in predictive performance between sex groups and among indicators. Among girl students, WWI had the highest predictive value, with an AUC of 0.73 (0.72–0.73), followed by BRI (0.72). Among boy participants, BRI (0.66) and WWI (0.65) performed better. Overall, WWI and BRI demonstrated the best predictive performance (both with an AUC of 0.69), followed by WC (0.65), while ABSI (0.60) and BMI (0.56) were relatively weaker. Specific AUC values are shown in Table 5.

**Table 5:** Relationship between various body composition measures and area under the curve of the 20-m SRT in Chinese adolescents aged 12–18.

Index	Boys (*n* = 22,719)	Girls (*n* = 23,198)	Total (*n* = 45,917)
BMI	0.57(0.56 ~ 0.58)	0.55(0.54 ~ 0.55)	0.56(0.55 ~ 0.56)
WC	0.63(0.62 ~ 0.64)	0.70(0.70 ~ 0.71)	0.65(0.65 ~ 0.66)
WWI	0.65(0.64 ~ 0.66)	0.73(0.72 ~ 0.73)	0.69(0.68 ~ 0.69)
ABSI	0.56(0.55 ~ 0.57)	0.64(0.63 ~ 0.65)	0.60(0.60 ~ 0.61)
BRI	0.66(0.65 ~ 0.67)	0.72(0.71 ~ 0.72)	0.69(0.68 ~ 0.69)

**Figure 2:**
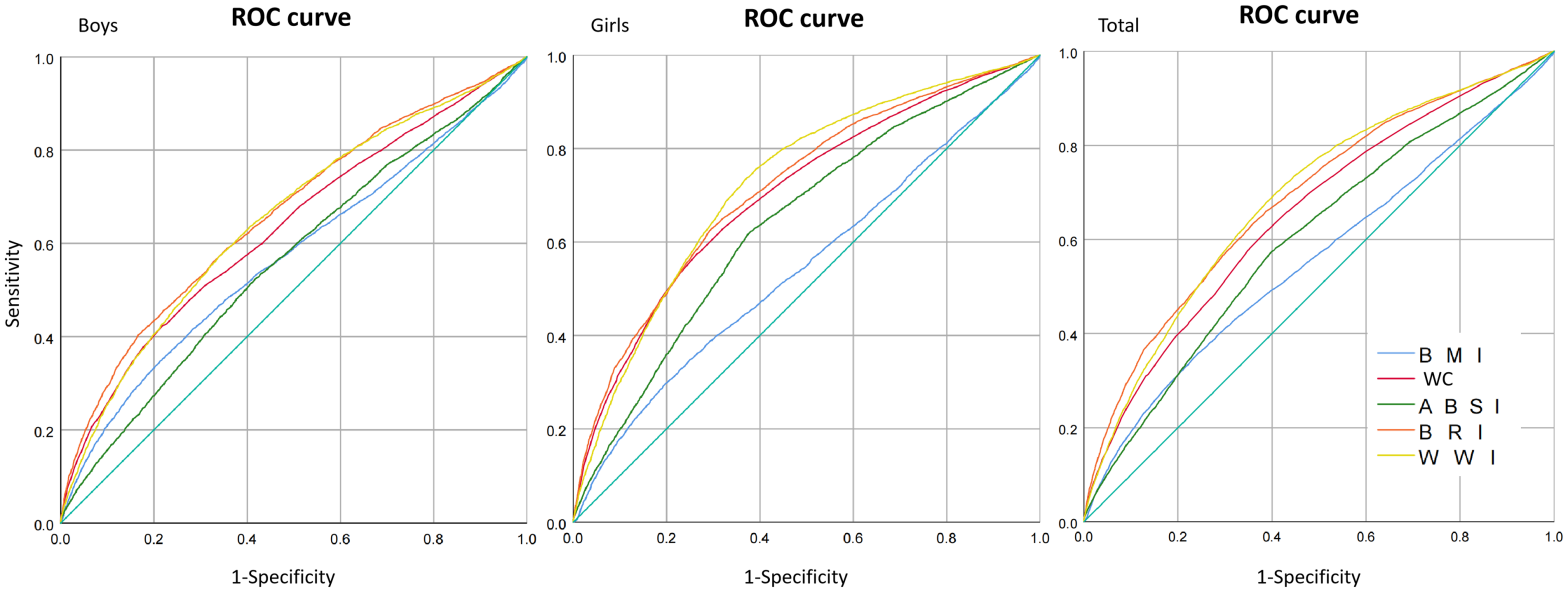
Schematic diagram illustrating the analysis of various body composition indices and the area under the 20-m SRT curve among Chinese adolescents aged 12–18.

Figure 2 shows the trends in AUC values for various body composition indices (BMI, WC, WWI, ABSI, and BRI) in predicting cardiorespiratory fitness among Chinese adolescents. Overall, it can be observed that the area under the curve for BMI is lowest among both boys and girls. The area under the curve for WWI is higher among girls than among boys.

## 4 Discussion

This study used a nationwide sample to analyze the associations between BMI, WC, WWI, ABSI, and BRI and cardiopulmonary fitness indicators in Chinese adolescents. The results indicate that BMI, WC, WWI, ABSI, and BRI in Chinese adolescents are significantly negatively correlated with the 20-m SRT, a measure of cardiorespiratory fitness. This suggests that body fat accumulation in Chinese adolescents is associated with poorer cardiorespiratory fitness performance, a finding consistent with previous research (40, 41). Further ROC analysis showed that the AUC values for the WWI and BRI indices among Chinese adolescents were 0.69, indicating that these indicators possess significant discriminatory power in identifying insufficient cardiorespiratory fitness. However, the analysis also showed that, based on the AUC values, there were significant sex differences in the relationship between various body composition indicators and cardiorespiratory fitness. Among Chinese adolescent girls, WWI had the highest predictive value, with an AUC of 0.73, followed by BRI at 0.72, whereas among boys, BRI (0.66) and WWI (0.65) demonstrated superior predictive power for identifying cardiopulmonary fitness. Overall, it can be observed that WWI and BRI demonstrated the best predictive performance for identifying insufficient cardiorespiratory fitness in Chinese adolescents, followed by WC, while BMI showed the weakest performance. This indicates that there are significant sex differences in the ability of different body composition indices to predict cardiorespiratory fitness, which warrants attention and further research.

The results of this study indicate that, overall, the WWI and BRI are the most effective indicators for assessing the cardiorespiratory fitness levels of Chinese adolescents. There are several reasons for this. First, the WWI and BRI take into account factors such as body weight and WC, thereby providing a more accurate and comprehensive reflection of abdominal fat accumulation in adolescents, and there is a significant correlation between abdominal fat accumulation and cardiorespiratory fitness (42). A study found that BRI is a better indicator of abdominal fat in adolescents; there is a significant correlation between BRI and health indicators, making it a key new indicator for assessing physical health (43). Other studies have shown that the WWI, which combines body weight and waist circumference, provides a better reflection of abdominal fat levels in adolescents and is closely associated with the risk of all-cause mortality (44). Second, the results of this study indicate that, overall, BMI is the least effective measure for identifying insufficient cardiorespiratory fitness among Chinese adolescents. This may be because, although BMI reflects body composition, it does not incorporate WC indicators in its analysis; consequently, it fails to adequately reflect abdominal fat, leading to certain limitations in its association with and validity for assessing cardiorespiratory fitness. Research indicates that compared to BMI, WWI better reflects abdominal fat content. In terms of its relationship with health indicators, WWI demonstrates significantly greater predictive power than BMI (45). This study used the 20-m SRT as an indirect measure of cardiopulmonary fitness among Chinese adolescents. During the assessment, excessive body weight requires the body to consume more oxygen and overcome greater resistance due to body mass, thereby leading to a decline in cardiopulmonary fitness levels.

This study demonstrates a significant association between measures that better reflect abdominal fat and cardiorespiratory fitness, indicating a significant link between visceral fat and cardiorespiratory fitness. Previous research has also shown a stronger association between abdominal fat content—a component of body composition—and cardiorespiratory fitness (46). There are multiple factors at play here. First, the accumulation of abdominal fat releases large amounts of harmful adipokines and inflammatory cytokines, such as TNF-α, IL-6, and resistin, while also reducing the secretion of beneficial adiponectin. These inflammatory effects can impair cardiovascular health and metabolic function, leading to a decline in cardiorespiratory fitness (47). Research also shows that inflammation in the body can cause changes in the circulatory and cardiovascular systems, leading to a decline in cardiorespiratory fitness. Furthermore, the accumulation of visceral fat can result in the abnormal deposition of fat in the visceral region, the liver, and skeletal muscles, leading to a decline in muscle function and, consequently, a reduction in cardiorespiratory fitness (48).

It is worth noting that in this study, the BRI was most effective in identifying insufficient cardiorespiratory fitness among Chinese adolescent boys, whereas the WWI was most effective among girls, indicating a certain degree of sex difference. These findings suggest that in future efforts to identify insufficient cardiorespiratory fitness, different analytical indicators should be used based on sex, boys tend to use the BRI metric, while girls tend to use the WWI metric for identification. The study shows that the BRI estimates an individual’s body roundness by combining height and WC, and theoretically better reflects visceral fat accumulation; whereas the WWI is defined as the ratio of WC to the square root of body weight, emphasizing changes in WC while controlling for the influence of body weight, and is considered to provide a purer measure of central obesity (49, 50). At the same time, girls place greater emphasis on the aesthetic appearance of their bodies than boys do; in particular, girls are stricter and more concerned about controlling their WHR (51). This may be the primary reason why WWI was more effective in identifying insufficient cardiorespiratory fitness among girls than among boys in this study. Furthermore, compared to BMI, which reflects overall obesity, indicators of central obesity that reflect abdominal fat distribution—particularly WWI and BRI—are more sensitive in identifying insufficient cardiorespiratory fitness in adolescents. This trend is particularly pronounced among adolescent girls, which may be related to the characteristics of body fat distribution in adolescent girls and the impact of hormonal changes on metabolic function (52). The results of this study also indicate that BMI is the least effective measure for identifying poor cardiorespiratory fitness. This may be because BMI is calculated solely based on height and weight and does not adequately reflect abdominal fat, resulting in its limited ability to identify poor cardiorespiratory fitness. Research shows that there is a more significant association between abdominal fat accumulation and cardiorespiratory fitness in adolescents (53).

To our knowledge, this study is the first to use a nationwide sample to analyze the association between BMI, WC, WWI, ABSI, and BRI in Chinese adolescents and insufficient cardiopulmonary fitness. The findings may provide valuable references and insights for future efforts to improve cardiopulmonary fitness and develop intervention strategies for Chinese adolescents. However, this study also has certain limitations. First, this study is a cross-sectional study, which can only analyze cross-sectional associations and cannot determine causal relationships. Future prospective cohort studies should be conducted to analyze the causal relationships between various body composition indices and cardiopulmonary fitness. Second, this study defined cardiopulmonary fitness insufficiency as a score below one standard deviation, which may introduce some bias compared to actual conditions and could also affect the analysis results. Future studies should adopt a more appropriate method for defining insufficient cardiopulmonary fitness to ensure more accurate analysis of the research findings.

## 5 Conclusion

This study found that both the WWI and BRI demonstrated equivalent predictive validity in identifying cardiovascular fitness deficits among Chinese adolescents. The BRI was most effective for boys, while the WWI was most effective for girls, indicating a sex difference. In the future, the WWI and BRI can serve as important indicators for identifying cardiovascular fitness deficits among Chinese adolescents, thereby better promoting improvements in cardiovascular fitness levels and the implementation of intervention measures, and providing valuable recommendations and references for the healthy development of Chinese adolescents.

## Data Availability

The raw data supporting the conclusions of this article will be made available by the authors, without undue reservation. Requests to access these datasets should be directed to lixiongon_112@126.com.
